# Supplementary material for: Intradermal Immunization of SARS-CoV-2 Original Strain Trimeric Spike Protein Associated to CpG and AddaS03 Adjuvants, but Not MPL, Provide Strong Humoral and Cellular Response in Mice
Source: Vaccines (Basel). 2022 Aug 12;10(8):1305. doi: 10.3390/vaccines10081305 (PMC9415730; doi:10.3390/vaccines10081305)
Supplement: Supplementary file 1 [file vaccines-10-01305-s001.zip › vaccines-1813895-supplementary.pdf]

vaccines

MDPI

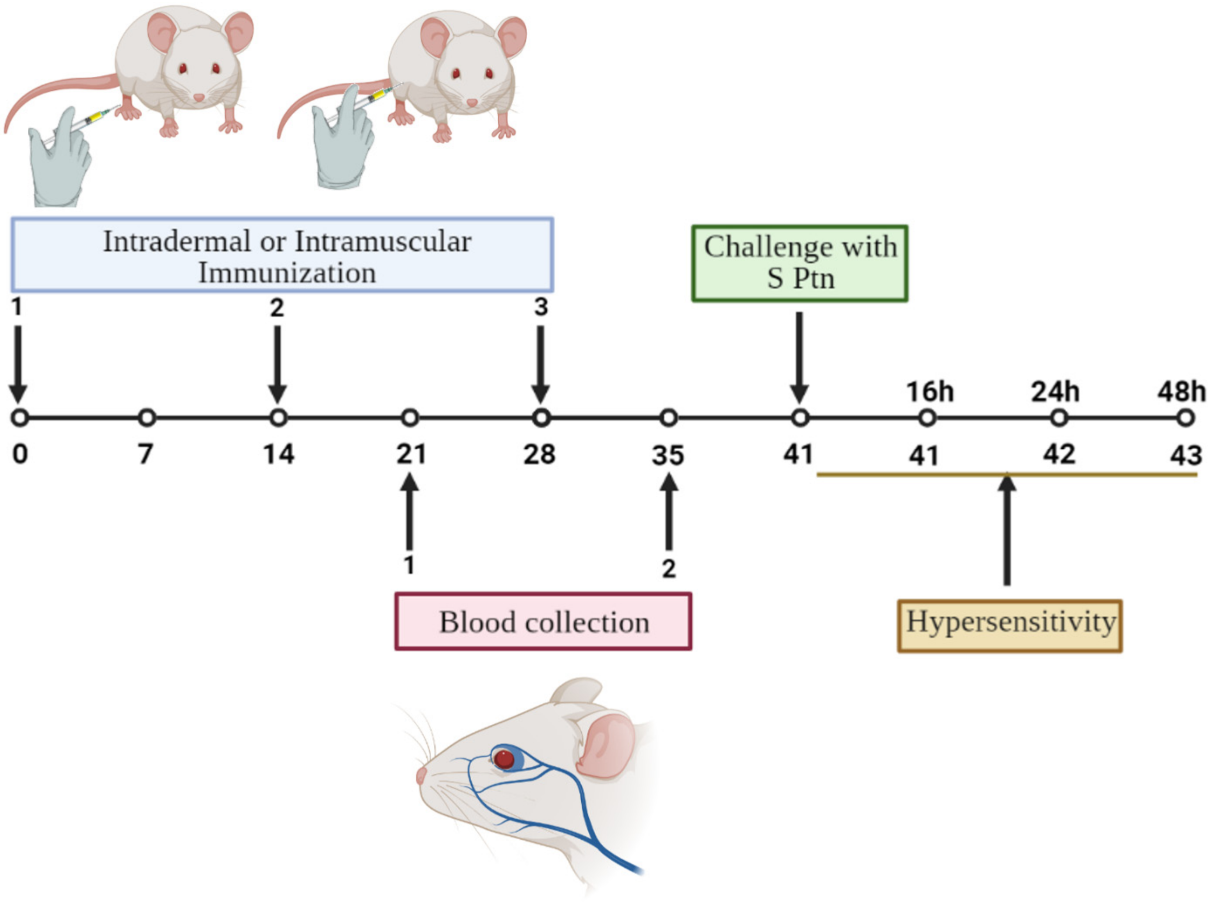

Figure S1: Experiments timeline**:** Timeline representing immunization, blood collection, challenge, and euthanasia days. Figure was made using the BioRender website and Microsoft PowerPoint.

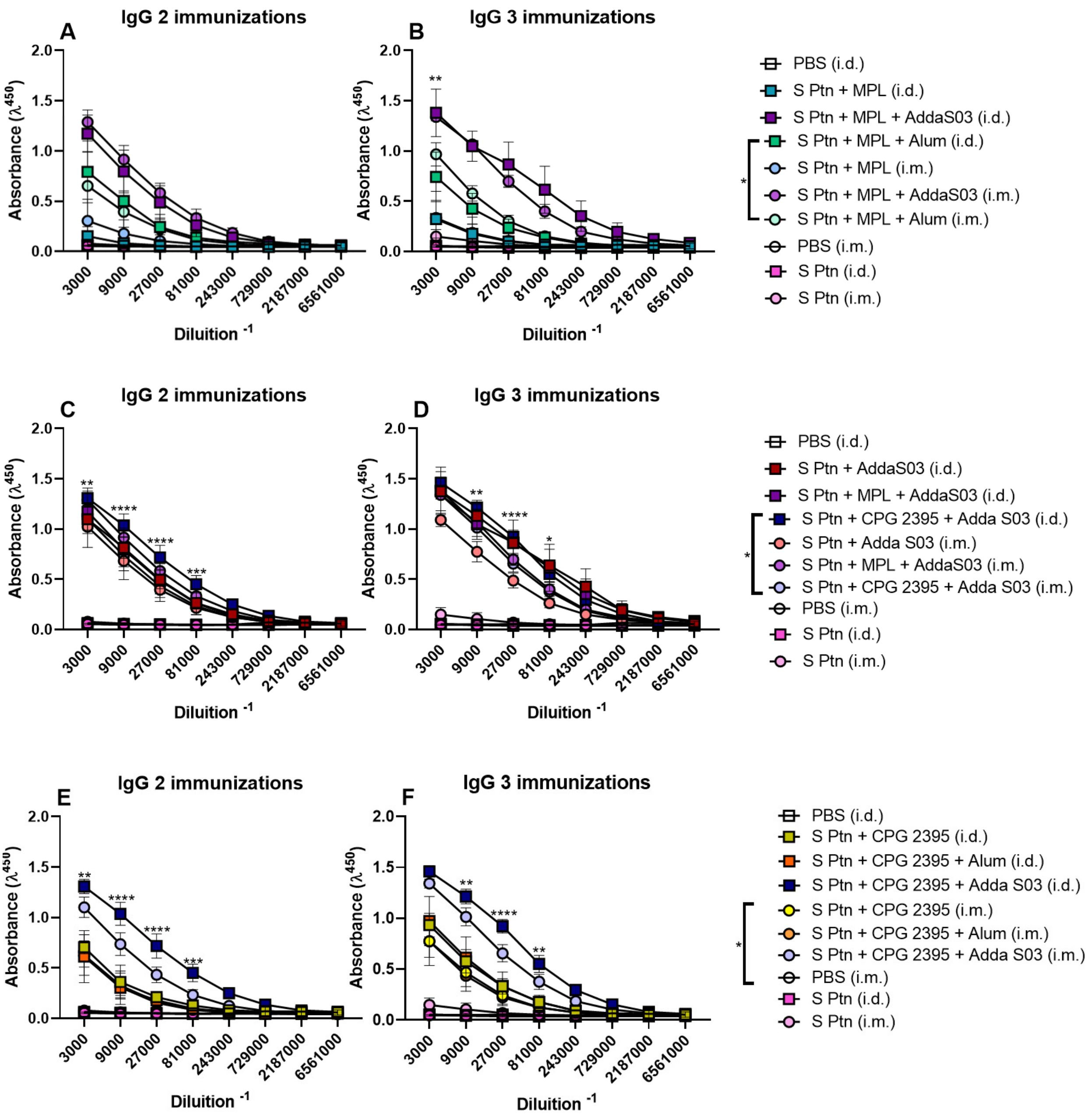

Figure S2: IgG curves after the first and second boosts: Mice were immunized with a 14-day interval between doses and blood samples were taken 7 days after each immunization. The serum levels of IgG were assessed by ELISA after 2 (A,C,E) and 3 (B,D,F) immunizations. Differences between the i.d. and i.m. route are shown. * $p < 0.05$; ** $p < 0.01$; *** $p < 0.001$; **** $p < 0.0001$. Two-way ANOVA. Normalized data from three independent experiments are shown. The S Ptn i.m. (n = 5), S Ptn i.d. (n = 5), S ptn + CPG 2395 i.m. (n = 5), S ptn + MPL i.m. (n = 5), S ptn + AddaS03 i.m. (n = 5), S ptn + MPL + Alum i.m. (n = 5), S ptn + CPG 2395 + Alum i.m. (n = 5), S ptn + CPG 2395 + AddaS03 i.m. (n = 5), S ptn + CPG 2395 i.d. (n = 5), S ptn + MPL i.d. (n = 5), S ptn + MPL + AddaS03 i.d. (n = 5), S ptn + MPL + Alum i.d. (n = 5), S ptn + CPG 2395 + Alum i.d. (n = 5) and S ptn + CPG 2395 + AddaS03 i.d. (n = 5) groups were assessed in one experiment. The PBS i.m. (n = 8), and S ptn + AddaS03 i.d. (n = 10) groups were assessed in two independent experiments. The PBS i.d. (n = 10) group was assessed in three independent experiments.

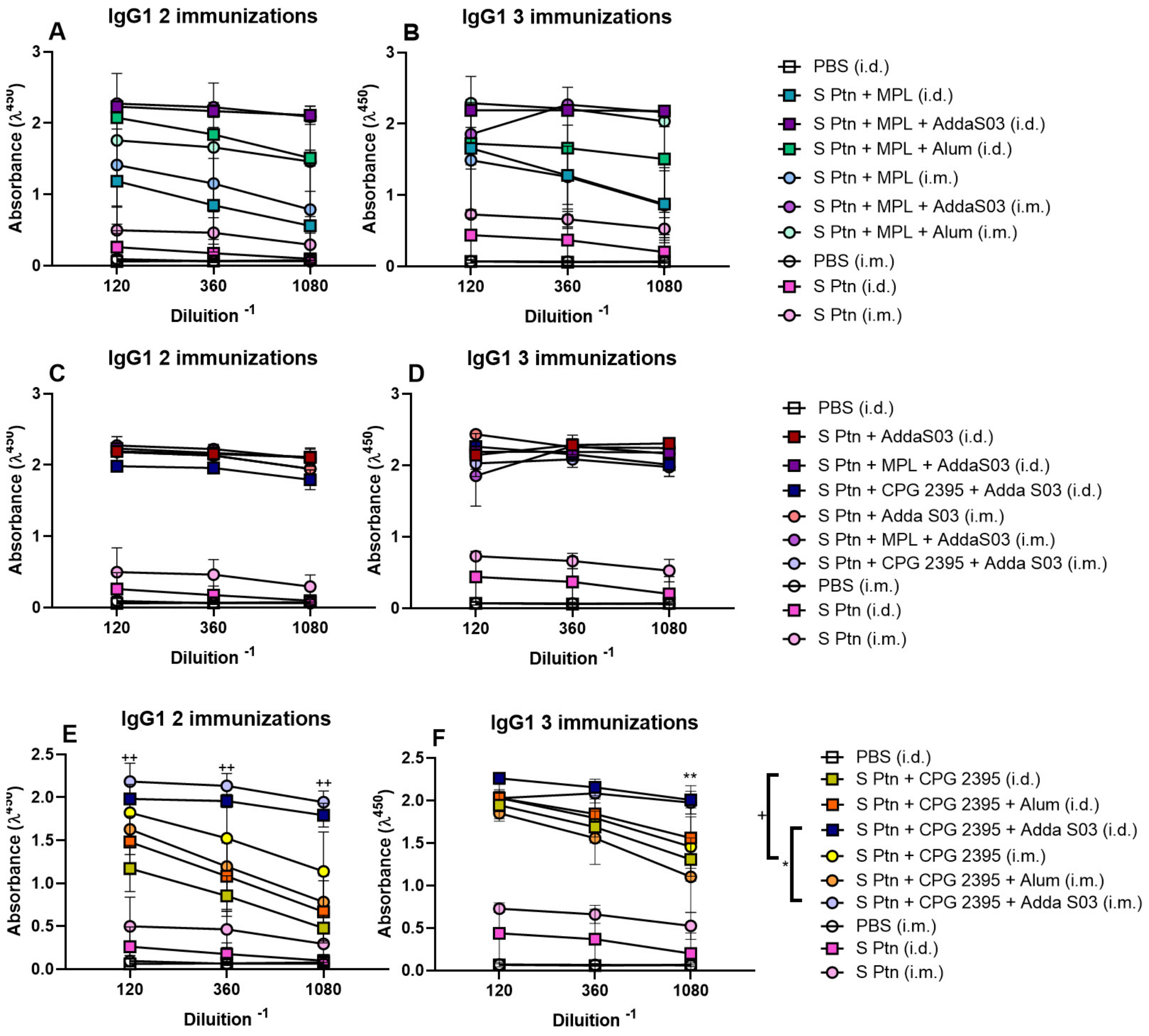

Figure S3: IgG1 curves after the first and second boosts: The mice were immunized with a 14-day interval between doses and blood samples were taken 7 days after each immunization. The serum levels of IgG1 were assessed by ELISA after 2 (A,C,E) and 3 (B,D,F) immunizations. Differences between the i.d. and i.m. routes are shown. ** or ++ $p < 0.01$. Two-way ANOVA. Normalized data from three independent experiments are shown. The S Ptn i.m. (n = 5), S Ptn i.d. (n = 5), S ptn + CPG 2395 i.m. (n = 5), S ptn + MPL i.m. (n = 5), S ptn + AddaS03 i.m. (n = 5), S ptn + MPL + Alum i.m. (n = 5), S ptn + CPG 2395 + Alum i.m. (n = 5), S ptn + CPG 2395 + AddaS03 i.m. (n = 5), S ptn + CPG 2395 i.d. (n = 5), S ptn + MPL i.d. (n = 5), S ptn + MPL + AddaS03 i.d. (n = 5), S ptn + MPL + Alum i.d. (n = 5), S ptn + CPG 2395 + Alum i.d. (n = 5), and S ptn + CPG 2395 + AddaS03 i.d. (n = 5) groups were assessed in one experiment. The PBS i.m. (n = 8) and S ptn + AddaS03 i.d. (n = 10) groups were assessed in two independent experiments. The PBS i.d. (n = 10) group was assessed in three independent experiments.

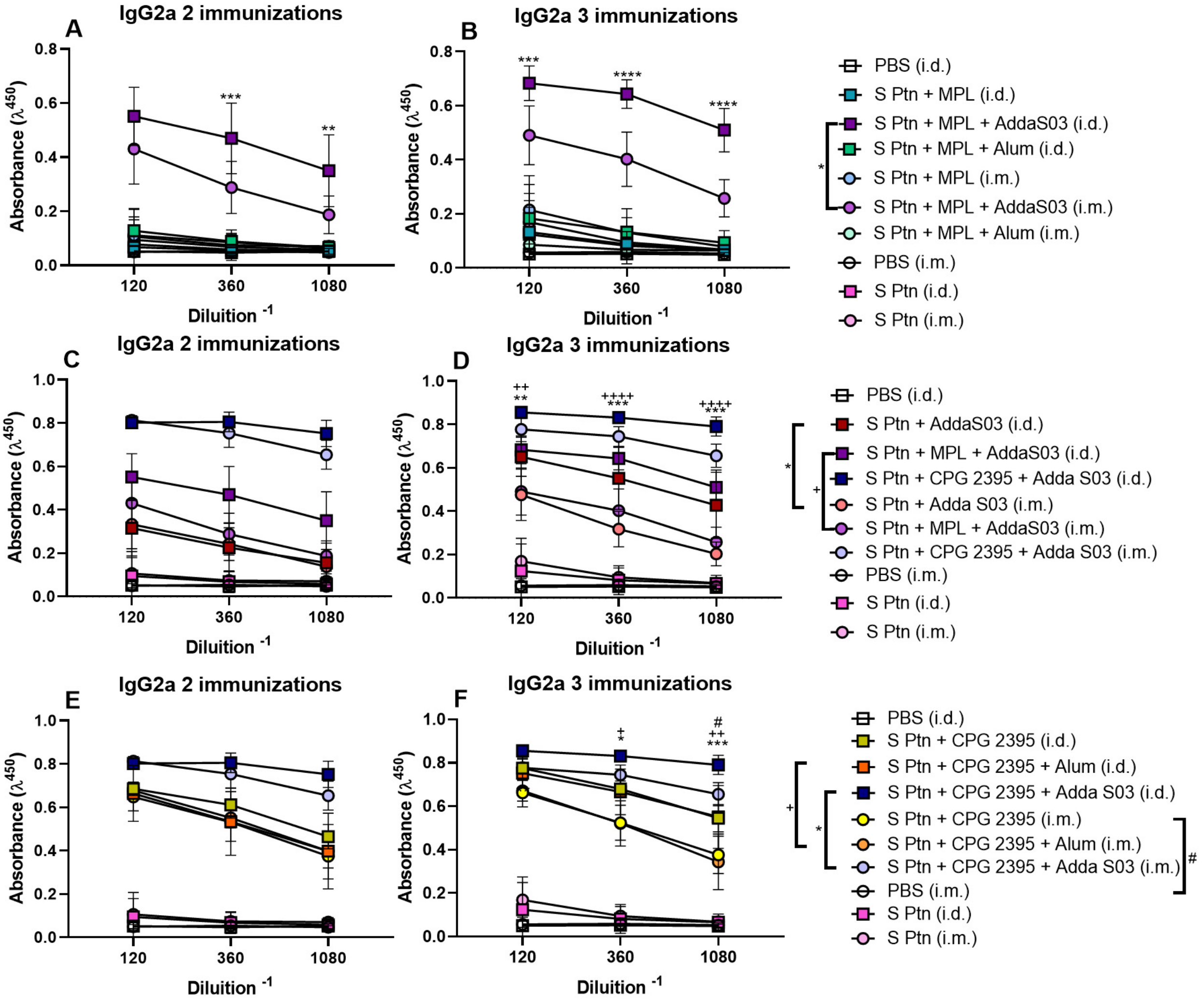

Figure S4: IgG2a curves after the first and second boosts: The mice were immunized with a 14-day interval between doses and blood samples were taken 7 days after each immunization. The serum levels of IgG2a were assessed by ELISA after 2 (A,C,E) and 3 (B,D,F) immunizations. Differences between the i.d. and i.m. route are shown. *, # or + $p < 0.05$; ** or ++ $p < 0.01$; *** $p < 0.001$; **** or ++++ $p < 0.0001$. Two-way ANOVA. Normalized data from three independent experiments are shown. The S Ptn i.m. (n = 5), S Ptn i.d. (n = 5), S ptn + CPG 2395 i.m. (n = 5), S ptn + MPL i.m. (n = 5), S ptn + AddaS03 i.m. (n = 5), S ptn + MPL + Alum i.m. (n = 5), S ptn + CPG 2395 + Alum i.m. (n = 5), S ptn + CPG 2395 + AddaS03 i.m. (n = 5), S ptn + CPG 2395 i.d. (n = 5), S ptn + MPL i.d. (n = 5), S ptn + MPL + AddaS03 i.d. (n = 5), S ptn + MPL + Alum i.d. (n = 5), S ptn + CPG 2395 + Alum i.d. (n = 5) and S ptn + CPG 2395 + AddaS03 i.d. (n = 5) groups were assessed in one experiment. The PBS i.m. (n = 8) and S ptn + AddaS03 i.d. (n = 10) groups were assessed in two independent experiments. The PBS i.d. (n = 10) group was assessed in three independent experiments.
